# Improving Pyroelectric Energy Harvesting Using a Sandblast Etching Technique

**DOI:** 10.3390/s130912113

**Published:** 2013-09-10

**Authors:** Chun-Ching Hsiao, An-Shen Siao

**Affiliations:** Department of Mechanical Design Engineering, National Formosa University, No. 64, Wunhua Rd., Huwei Township, Yunlin County 632, Taiwan, E-Mail: qbasic147@gmail.com

**Keywords:** pyroelectricity, thermal energy, temperature variation rate, sandblast etching, energy harvesting

## Abstract

Large amounts of low-grade heat are emitted by various industries and exhausted into the environment. This heat energy can be used as a free source for pyroelectric power generation. A three-dimensional pattern helps to improve the temperature variation rates in pyroelectric elements by means of lateral temperature gradients induced on the sidewalls of the responsive elements. A novel method using sandblast etching is successfully applied in fabricating the complex pattern of a vortex-like electrode. Both experiment and simulation show that the proposed design of the vortex-like electrode improved the electrical output of the pyroelectric cells and enhanced the efficiency of pyroelectric harvesting converters. A three-dimensional finite element model is generated by commercial software for solving the transient temperature fields and exploring the temperature variation rate in the PZT pyroelectric cells with various designs. The vortex-like type has a larger temperature variation rate than the fully covered type, by about 53.9%.The measured electrical output of the vortex-like electrode exhibits an obvious increase in the generated charge and the measured current, as compared to the fully covered electrode, by of about 47.1% and 53.1%, respectively.

## 1. Introduction

Waste heat refers to heat produced by lights, traffic vehicles, machines, electrical equipment and industrial processes; it has no useful application. Energy is often produced by a heat engine working on a source of high-temperature heat. According to the second law of thermodynamics, a heat engine can never have perfect efficiency. Therefore, waste heat is regarded as a waste by-product of this process; however, it can be a rich source of energy through the harvesting of this thermal energy [1–3]. Large amounts of low temperature waste heat discharged into the surroundings are the by-product of air conditioning, refrigeration and heat pump apparatuses. A large part of this energy resource consumption is lost, mainly in the form of low-temperature waste heat. The potential for waste heat recovery includes various sources: heat in flue gases, heat in vapor streams, convective and radiant heat lost from the exteriors of equipment, *etc.* As various types of waste heat are discharged into the environment, patterns, distributions, densities, magnitudes and intensities must be taken into consideration when designing pyroelectric harvesters [4].

Pyroelectricity is the appearance of an electric charge at the surface of a polar material when the temperature changes the polarization. Although artificial pyroelectric materials (PVDF [1], PZT [2], PMN/PT [5], PZN/PT [6], *etc.*) have been engineered, the pyroelectric effect was first discovered in minerals such as tourmaline. A pyroelectric charge in minerals develops on the opposite faces of asymmetric crystals. The direction in which the propagation of the charge tends toward is usually constant throughout a pyroelectric material, but in some materials this direction can be changed by a nearby electric field. On the other hand, non-uniform heating or cooling leads to an inhomogeneous distribution of thermal expansion in pyroelectric materials; this expansion generates non-uniform stresses. These stresses can result in polarization through a piezoelectric effect. This is called the tertiary pyroelectric effect [7]. Recently, pyroelectric materials, which make use of the pyroelectric effect to create a flow of charge to or from the surface of a material as a result of successive heating or cooling, have also been used for the conversion of thermal energy to electrical energy. In fact, a very small change in temperature can produce a pyroelectric current, a process which is used extensively in infrared detection for imaging and motion sensing, as well as for thermometry applications. Pyroelectric energy modules offer a novel energy conversion technology by transforming waste heat into electricity, which converts the time-dependent temperature variations into electrical energy. Pyroelectric energy conversion devices require the thermal cycling of a pyroelectric element between a hot and a cold temperature source in order to produce electricity [1–3,5]. The generated energy is harvested by delivering it to an external load or storage unit. The conversion ratio for pyroelectric energy harvesting is much larger; in theory, it can reach the conversion ratio of the Carnot cycle, whatever the material properties may be. However, the material of the fabrication properties limits the conversion ratio of the thermoelectric modules [5].

Pyroelectric materials have a spontaneous polarization in the absence of an applied electric field. The spontaneous polarization is largely dependent on temperature transmission through the crystallographic structure of the pyroelectric material. The pyroelectric effect is generated by the displacement of the atoms from their equilibrium positions upon heating and cooling. The electric current (i_p_) generated by pyroelectric materials is given by [2,8]:

(1) $${\text{i}}_{\text{p}}=\text{dQ}/\text{dt}=\eta \times \lambda \times \text{A}\times \text{dT}\;/\text{dt}$$

where Q is the induced charge, η is the absorption coefficient of radiation, λ is the pyroelectric coefficient, A is the surface area of the pyroelectric material and dT/dt is the heating or cooling rate. At steady state (dT/dt = 0), the polarization is constant and no current is generated. However, a rise in temperature (dT/dt > 0) reduces the overall polarization through a reduction in the dipole moment. The number of bound charges decreases, and the subsequent redistribution of charges results in the current flowing in the circuit. If a pyroelectric material is cooled (dT/dt < 0), the direction of the current is reversed. In an open circuit, the free charges remain on the electrodes and a voltage is established between them. The direction of polarization is usually constant throughout a pyroelectric material, but in some materials this direction can be changed by applying a coercive poling electric field. The pyroelectric coefficient (λ) is given by [2,8]:

(2) $$\lambda =\text{d}{\text{P}}_{\text{s}}/\text{dT}$$

where P_s_ is the magnitude of the electrical polarization vector. Pyroelectric cells are sandwiched between a top and a bottom electrode, as flat-plate capacitors, and poled along the axis perpendicular to the plates. The magnitude of P_s_ is equal to the electrode charge density, while P_s_ is perpendicular to the electrode surface. The temperature variation rate is a critical factor in enhancing the electric response of pyroelectric devices when pyroelectric materials and geometry are determined. Increasing the temperature variation rates in pyroelectric cells leads to a higher response current in the devices.

## 2. Background and Motives

A partially covered top electrode has a higher responsivity than a fully covered top electrode does because the uncovered part of the ZnO layer is directly exposed to the heat source and, thus, markedly increases the heat absorption [9]. Although increasing the bare electrode area can improve the heat absorption of pyroelectric materials and the temperature variation rate, the generated charges decrease due to the smaller top electrode area's ability to hold an electrical charge. Conversely, increasing the top electrode area can increase the generated charge, as the temperature variation rates decrease due to the heat absorption of the pyroelectric materials blocked from the fully covered electrode. Therefore, the design of pyroelectric devices needs to monitor both the temperature variation rate and the electrode area. Moreover, a three-dimensional pattern of the responsive element of LiTaO_3_ has been used to enhance the temperature variation rate through lateral temperature gradients induced on the sidewalls of the responsive element under homogeneous irradiation [10]. By adopting the concepts of both the mesh electrode and the three-dimensional pattern, the PZT sheet is further etched to produce deeper cavities and a smaller electrode width. This induces lateral temperature gradients on the sidewalls of cavities, thereby enhancing the temperature variation rate under homogeneous heat irradiation, although PZT etchant with a low etching rate is unsuitable for creating the deeper cavities in the PZT sheet [11]. Developing a manufacturing method which creates these deeper cavities with a smaller electrode width in a thicker PZT sheet was an arduous task. A straight trench structure in PZT sheets was conceived for digging deeper cavities by a precision dicing saw apparatus. The width of the trenches was controlled by a hub blade, and the depth of the trenches was determined and controlled by the dicing saw machine. While trenching the PZT sheets to produce deeper trenches effectively enhanced the temperature variation rate in a thicker PZT pyroelectric cell as a result of lateral temperature gradients induced by the trenched electrode, a complex pattern fitting various distributions, densities, magnitudes and intensities of waste heat sources was difficult to fabricate using a precision dicing saw [12]. A run-of-the-mill straight trench structure manufactured by the dicing saw machine cannot fit a variety of waste heat sources for enhancing thermal energy harvesting. A pattern design in PZT sheets was a prerequisite for addressing the issue of various waste heat sources, including their distributions, densities, magnitudes and intensities. Hence, creating a complex pattern resulting in deeper trenches in PZT sheets was imperative in order to enhance the temperature variation rate and the efficiency of pyroelectric harvesting converters. The designs of patterns and trenches needed to take into consideration gas flow modes, routes and rates in the PZT sheets in order to enhance the heat absorption and the temperature variation rate under a waste heat recovery system of heated gas exhaust. In other words, increasing the heated gas within the PZT sheets would improve the heat absorption from the heated gas and enhance the temperature variation rate.

The Olsen cycle enabled the generation of significant electrical energy using the pyroelectric effect. Nguyen *et al.* [3] used dipping experiments to perform the Olsen cycle using a piston oscillating vertically and driving silicon oil back and forth between a heat source and a cold heat exchanger in a Teflon cylindrical chamber. Furthermore, Lee *et al.* [1] used stamping experiments to implement the Olsen cycle by alternately placing a pyroelectric material for heat conduction with a cold and a hot source. The different heat transfers were also shown to affect the efficiency of the Olsen cycle. In addition, Cuadras *et al.* [2] used air currents to generate the heating and cooling temperature fluctuations applied to the pyroelectric converters. Current from the cyclic temperature fluctuations was rectified and stored in a 1 μF capacitor as a storage element, which was sufficient to power typical autonomous sensor nodes. A pattern design in PZT sheets for fitting various heat sources was necessary to improve the temperature variation rates. Creating a complex pattern with deeper trenches made in the PZT sheets presented a considerable challenge. The present study investigated the use of sandblast etching to produce three-dimensional complex patterns in PZT pyroelectric cells to enhance the performance of pyroelectric harvesting converters. The three-dimensional complex patterns with deeper trenches in the PZT pyroelectric cells were compared to prove the effect of these patterns on the efficiency of PZT pyroelectric harvesters under a waste heat mode of heated gas exhaust.

## 3. Experiments and Methods

### 3.1. Design

**Figure 1.**
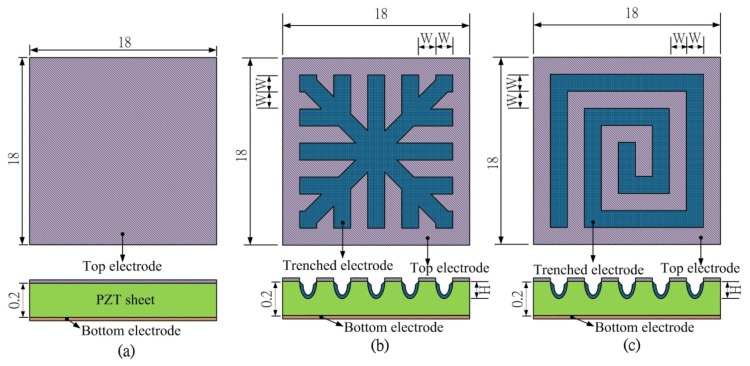
Top electrode dimensions in mm: (**a**) Fully covered electrode; (**b**) Branch-like electrode; (**c**) Vortex-like electrode.

A PZT pyroelectric cell with the dimensions of 18 mm × 18 mm × 0.214 mm was used; it comprised a 0.2 mm thick PZT sheet sandwiched between a top and a bottom electrode. A 7 μm thick silver film was used for the electrodes. PZT samples were provided by Eleceram Technology Co. (Taiwan) Table 1 shows the properties of the commercial PZT pyroelectric cell. Although commercial PZT bulk material possesses excellent pyroelectric properties, a thick pyroelectric material with a high thermal capacity can obstruct quick temperature variations. Moreover, etching and trenching the PZT sheets using etchants and a dicing saw apparatus have some drawbacks for further applications due to the low etching rate and the run-of-the-mill straight trench structure [11,12]. The use of a dicing saw apparatus to trench the PZT sheets makes it possible to dig deeper trenches [12], but the run-of-the-mill straight trench structure cannot be easily fitted to various types and distributions of waste heat sources discharged to the environment, such as heated gas exhaust. Therefore, creating a complex pattern made in the PZT sheets was critical to enhancing the performance of pyroelectric converters. Sandblast etching is a novel fabrication method for producing a complex pattern in the PZT sheets. Three patterns were fabricated in the PZT sheets: fully covered, branch-like and vortex-like electrodes, as shown in Figure 1. Furthermore, the trench width (W) was fixed at 100 μm, while the trench depth (H) was controlled at 120 μm. These patterns were compared under a heated gas exhaust waste heat mode.

**Figure 2.**
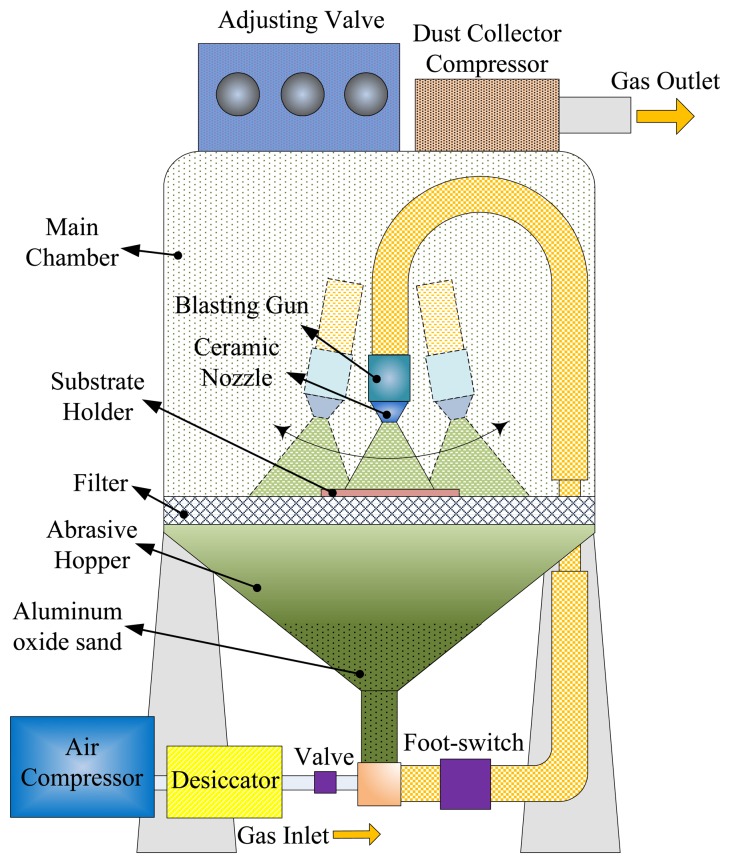
Schematic diagram of the sandblast etching apparatus.

The schematic diagram of the sandblast etching apparatus is shown in Figure 2. This machine, molded into an inhaled dry blasting machine, was manufactured and assembled by the Shang-Po Sander Co., Ltd. (Taiwan). An inhaling dry sandblasting machine generally consists of six systems: (i) the structural system, (ii) the media power system, (iii) the piping system, (iv) the dust removal system, (v) the control system and (vi) the auxiliary system. The working principle of sandblast etching is to use compressed air carrying aluminum oxide sand at high speed to collide with the surface of the workpiece; compressed air flows through a valve to form negative pressure, so aluminum oxide sand will be drawn into the blasting gun. Aluminum oxide sand is accelerated and spurted from the ceramic nozzle to etch substrates.

### 3.2. Modeling and Simulation

**Figure 3.**
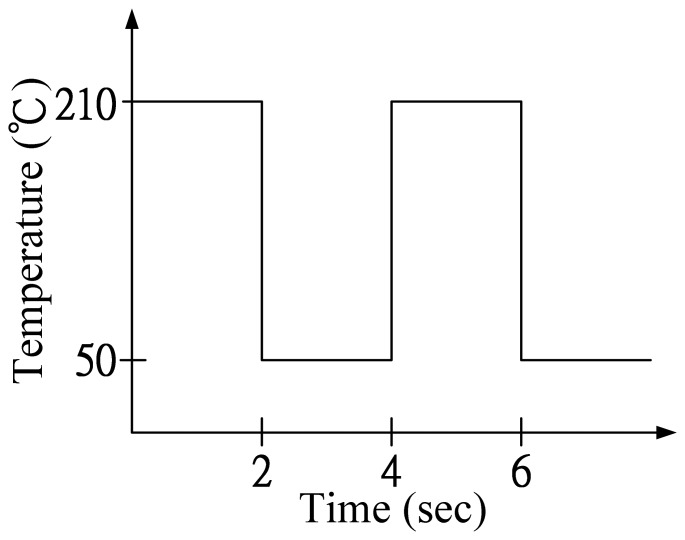
Square waveform used to modulate the temperature range.

**Figure 4.**
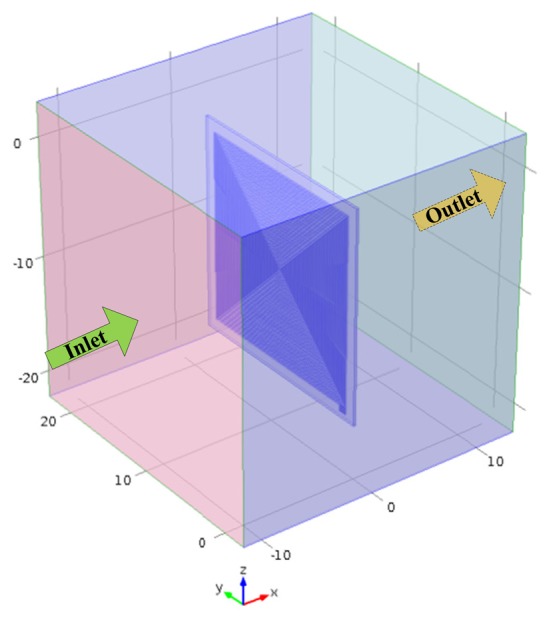
3D finite element model for the PZT pyroelectric cell (unit: mm).

The performance of pyroelectric cells can be estimated by means of the temperature variation rate of the pyroelectric sheets; however, extracting it is by no means an easy task. The difficulty is in extracting the temperature variation rate along the thickness direction of the pyroelectric sheets, and this temperature variation field in the sheets is not easy to extract by means of experimental measurements. In the present study, a three-dimensional finite element model was generated by commercial multiphysics software Comsol Multiphysics^®^ 4.2 to explore the temperature variation rate in the PZT pyroelectric cells with a fully covered electrode, as compared to a branch-like or a vortex-like electrode. The model solved the transient temperature fields for the PZT pyroelectric sheets and the air flow in a square channel, by using the heat transfer module. Thermal energy was transported through conduction in the PZT pyroelectric sheet, and through conduction and convection in a heating and cooling air flow. The temperature field was continuous across the internal surfaces between the PZT pyroelectric sheet and the air in the channel. A cube with a square cross-section of 25 mm × 25 mm and a depth of 25 mm was used to simulate the air flow channel, and a PZT pyroelectric sheet with dimensions of 18 mm × 18 mm × 0.2 mm was placed in the center of the cube. A temperature square waveform with an amplitude of 50 °C to 210 °C with a periodic time of 4 s (as shown in Figure 3) was applied on the lateral side of the cube as an inlet perpendicular to the trenched face of the PZT sheets with an air flow rate of about 0.5 m/s. The opposite side was an outlet, and the transport of thermal energy at the outlet was dominated by convection. The flow field was obtained by solving one momentum balance for each space coordinate and a mass balance. The inlet velocity was defined by a parabolic velocity profile for a fully developed laminar flow. At the outlet, a constant pressure was combined with the assumption that there were no viscous stresses in the direction perpendicular to the outlet. The thermal isolation condition was applied to the other sides of the cube. The thermal properties of the materials used in the model are shown in Table 2. There was an isotropic and time-independent assumption for the PZT pyroelectric sheet in the model. The thermal conductivity, heat capacity and air density were all temperature-dependent material properties, which were according to the setting in the Comsol software. The three dimensional FEM model (as shown in Figure 4) was meshed using a tetrahedral mesh.

### 3.3. Process

A structure consisting of deeper trenches would improve the temperature variation rate in PZT pyroelectric cells due to the lateral temperature gradients induced on the sidewalls of deeper trenches; these accelerate the heat absorption of pyroelectric cells [10]. However, deeper trenches with a complex pattern have proved hard to fabricate using traditional etching or dicing saw processes [11,12]. The aim of the novel fabrication technique using sandblast etching was to produce suitable patterns for fitting multiform waste heat discharges.

**Figure 5.**
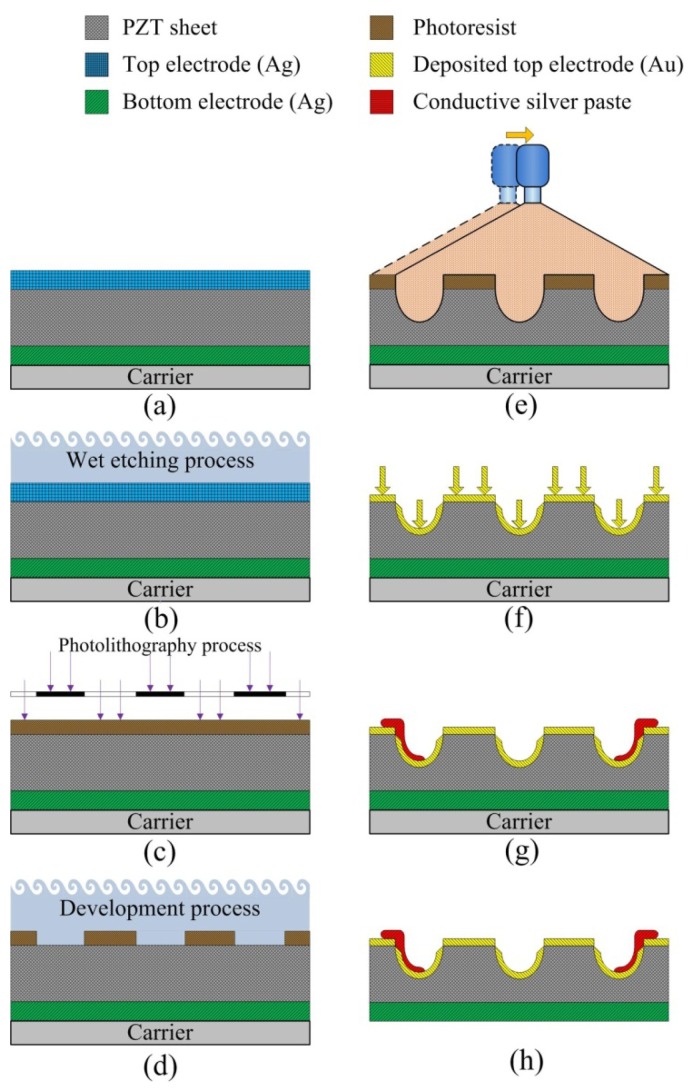
Process flow of the PZT pyroelectric cell.

**Figure 6.**
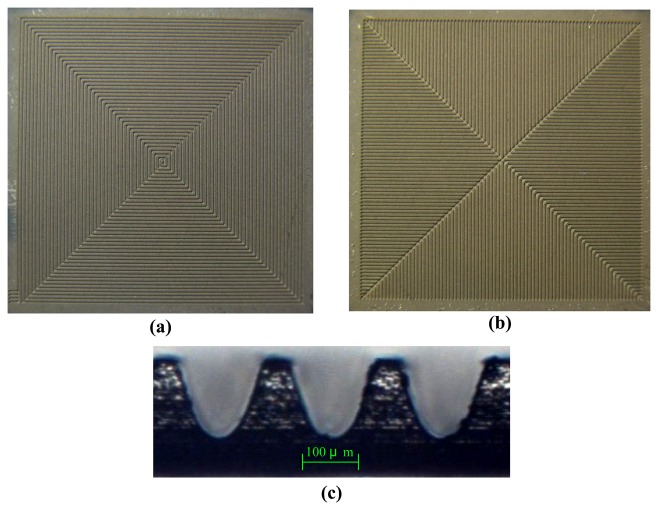
Fabricated pyroelectric cells: (**a**) vortex-like electrode; (**b**) branch-like electrode; (**c**) cross section of trenches.

The fabrication flow of the PZT pyroelectric cells is shown in Figure 5. The starting process was attaching the PZT sheet to a carrier of glass substrate, as shown in Figure 5a. Wet etchant of HNO_3_-H_2_O = 7:3 was used to remove the fully covered top electrode, as shown in Figure 5b. A Laminar^®^SB220 dry film with photoresist at a thickness of 0.075 mm was unfolded and attached to the PZT sheet manually, as shown in Figure 5c. This dry film, a negative-acting photoresist for the application of acid plating, tent-etch and print-etch, possessed features such as: excellent tenting behavior characteristics, excellent resolution and fine line adhesion, good contrast after exposure for easy inspection and excellent conformation capability. The dry film was also patterned and exposed to UV light with a mask. Patterning the dry film photoresist was done using a developmental process with a solution of Na_2_CO_3_-H_2_O = 1:99, as shown in Figure 5d. A sandblast etching apparatus was used to manufacture the branch-like and the vortex-like patterns, with trench structures consisting of a width (W) and a depth (H) in the PZT sheets, as shown in Figure 5e. A 100 nm thick gold film was deposited on the top side of the PZT sheets to produce the top and the trenched electrodes using an E-beam evaporator, as shown in Figure 5f. At the end of the trenches, silver paste was used to connect the top and the trenched electrode to increase the electrode area and improve the generated charges, as shown in Figure 5g. Finally, the carrier was removed to achieve the fabrication of the PZT pyroelectric cells, as shown in Figure 5h. The fabricated pyroelectric cells with the branch-like and the vortex-like electrodes had an electrode width of 100 μm and an etching depth of 120 μm, as shown in Figure 6.

### 3.4. Measurement

**Figure 7.**
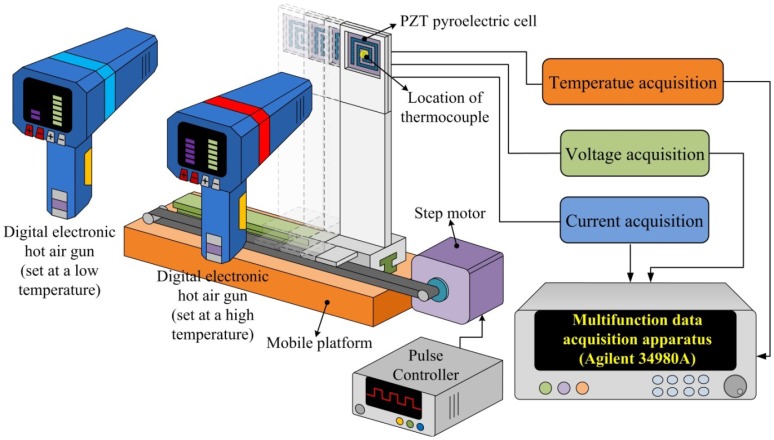
Schematic diagram of the integral measurement coupled thermal and electrical systems.

An integral measurement coupled thermal and electrical system, as shown in Figure 7, was set to estimate the performance of the present PZT cells. Two digital electronic hot air guns were used as thermal sources to control temperature fluctuations from 50 °C to 210 °C. Then, a mobile platform with a step motor controlled by a pulse controller was adopted to produce time-dependent temperature variations (dT/dt) in a period of 4 s. A fixture set on the mobile platform was used to hold the PZT cell along the edges in order to expose it completely to the air. The distance between the digital electronic hot air guns and the PZT cell was about 2 cm. The temperature in the PZT cell was measured using a thermocouple type K (Chromel/Alumel), which was attached to the bottom electrode at the center of the PZT cell ensuring good thermal contact. Finally, the output data of temperature, current and voltage were measured simultaneously with a computer-controlled data acquisition apparatus (Agilent 34980A).

**Figure 8.**
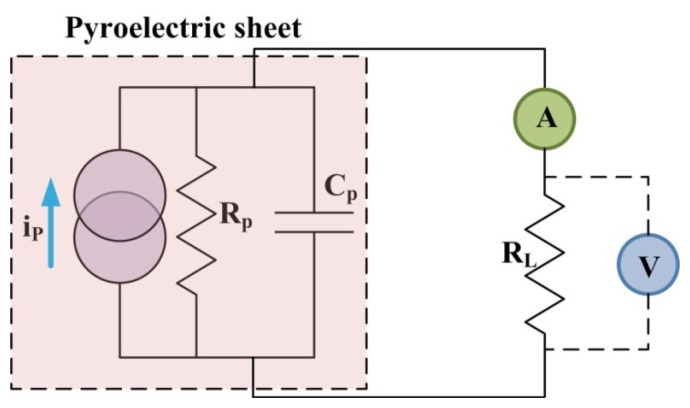
Circuit used to estimate the generated power of the PZT pyroelectric cells.

In addition, the PZT pyroelectric cells were further tested regarding expressions of both power and charge. The power was obtained by measured data of current as well as voltage under various load conditions (R_L_). A circuit used for power measurement is shown in Figure 8.

**Figure 9.**
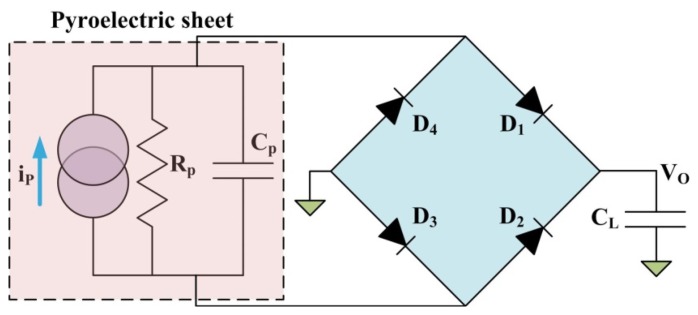
Full-wave rectifier circuit used to store the charge supplied with a pyroelectric sheet.

Moreover, the charge generated from thermal oscillations was stored in a capacitor C_L_ as a storage element using the full-wave rectifier circuit, as depicted in Figure 9. The measured forward voltage drop of the diodes (Model: 1N4148) was 0.62 V. C_L_ was a 22 μF electrolytic capacitor with a 50 V maximum voltage. During the heating cycle, the two forward-biased diodes (D_1_ and D_3_) allowed the generated current i_P_ flow through and charge C_L_. The other diodes (D_2_ and D_4_) were reverse-biased, and blocked the current flow. During the cooling cycle, the direction of i_P_ was reversed, and C_L_ was charged through D_2_ and D_4_. The temperature in the PZT sheets and the output voltage of the circuit (V_0_) were also measured with the data acquisition unit (Agilent 34980A).

## 4. Results and Discussion

### 4.1. Simulation Results

**Figure 10.**
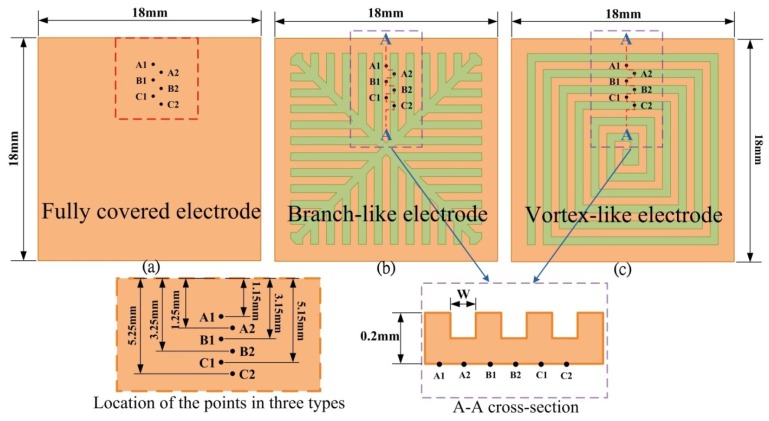
Points defined in the PZT sheets: (**a**) fully covered electrode; (**b**) branch-like electrode; (**c**) vortex-like electrode.

**Figure 11.**
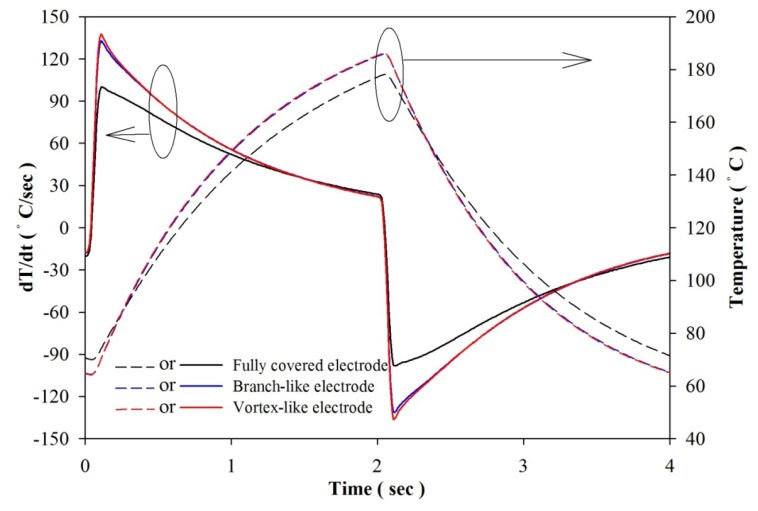
Relationship between temperature variation rate (dT/dt) and temperature *vs.* time in the PZT sheets at point A1 for the fully covered, branch-like and vortex-like electrodes.

**Figure 12.**
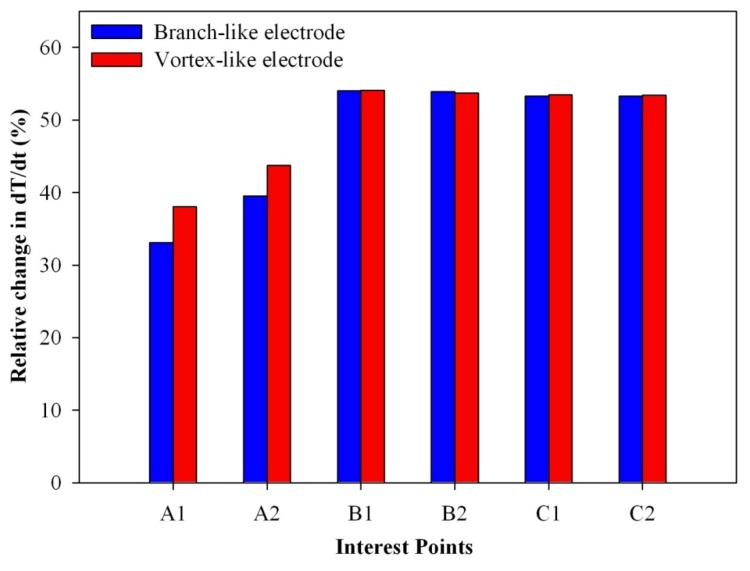
Relative change in the maximum peak value of the temperature variation rate at points A1, A2, B1, B2, C1 and C2 for the vortex-like and the branch-like types.

Transient temperature fields in the PZT pyroelectric sheets were simulated. Hsiao *et al.* [9,11,12] showed that the temperature variation rate in the PZT sheet increased gradually toward the top electrode as a result of the incidental radiation power applied to the top electrode. Points at the top side of the PZT sheets had the largest temperature variation rate, which was hard to improve. However, the temperature variation rate at the bottom of the PZT sheets was the lowest. An increase in the temperature variation rate at the point approaching the bottom side of the PZT sheets could certainly improve the efficiency of PZT pyroelectric energy converters. Interest points (A1, A2, B1, B2, C1 and C2) at the bottom of the PZT sheets were extracted to analyze the temperature variation rates for various designs, namely the fully covered, the branch-like and the vortex-like types. These points, as defined in Figure 10, were used to interpret the temperature variation rates in the PZT pyroelectric sheets. Points C1 and C2 were near the center of the PZT sheets, and points A1 and A2 were somewhat removed from the center of the PZT sheets. Points A2, B2 and C2 were placed under the trenched electrode, and points A1, B1 and C1 were placed under the top electrode. Figure 11 shows the temperature variation rate and temperature versus time curves in the period of 4 s at point A1 for various designs. When the PZT sheets acquired the incident heat energy, the temperature rapidly increased and the temperature variation rate instantly reached the maximum value. Then, the temperature variation rate declined due to the PZT sheets tending to thermal equilibrium. The relative change in dT/dt is defined as:

(3) $$\text{dT}/\text{dt}\;(\%)=[{\left(\text{dT}/\text{dt}\right)}_{\text{trench}}-{\left(\text{dT}/\text{dt}\right)}_{\text{primitive}}]/{\left(\text{dT}/\text{dt}\right)}_{\text{primitive}}\times 100\%$$

where (dT/dt)_trench_ is the maximum peak value of the temperature variation rate for the vortex-like or the branch-like type, and (dT/dt)_primitive_ is the maximum peak value of the temperature variation rate for the fully covered type. Figure 12 shows the relative change in the maximum peak value of the temperature variation rate at points A1, A2, B1, B2, C1 and C2 for the vortex-like and the branch-like types. When the points were near the center of the PZT sheets, the vortex-like and the branch-like types had a higher relative change in dT/dt. This could be attributed to the trench structure in the PZT sheets. When the points were somewhat removed from the center of the PZT sheets, the vortex-like type had a larger temperature variation rate than the branch-like type owing to the pattern design. Hence, the pattern designs with a deep trench structure obviously affected the transient temperature fields in the PZT pyroelectric sheets.

### 4.2. Experimental Results

**Figure 13.**
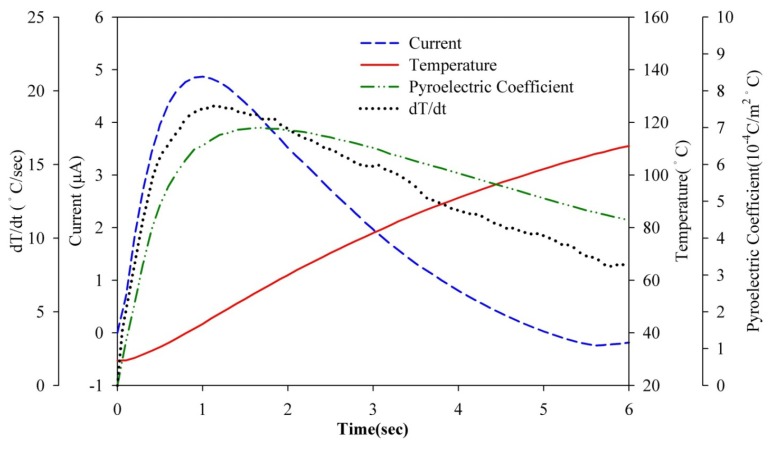
Pyroelectric coefficient, temperature, current *vs.* time curves for the PZT sheet with the fully covered electrode.

In order to precisely measure the pyroelectric coefficient, the PZT sheets were those using the original samples with the fully covered electrode provided by Eleceram Technology Co. The apparatus for measuring the pyroelectric coefficient, as shown in Figure 7, was used to record the output data for the pyroelectric current, the sample temperature versus time. Using the charge integration technique [2,13], the digital hot air gun acted as a heater with the mobile platform driven by a step motor controlled by the pulse controller to produce time-dependent temperature variations. The type K thermocouple (Chromel/Alumel) used to measure the temperature in the PZT sheets was attached to the PZT sheets to ensure a good thermal contact. The effective electrode area was 324 mm^2^. The output data of the temperature and current were measured simultaneously by a computer-controlled data acquisition system (Agilent 34980A). The charge was inferred from the integration of the area under current curves. Measurements of temperature variation and current yielded the pyroelectric coefficient. The generated charge is driven by integrating Equation (1) over time:

(3) $$Q=\int_{t_{i}}^{t_{f}}i_{p}dt=\int_{T_{i}}^{T_{f}}\lambda \times \text{AdT}=\lambda \times A\times (T_{f}-T_{i})$$

where *T_f_* and *T_i_* are, respectively, the temperature at the considered final (*t_f_*) and initial (*t_i_*) time. The generated charge depended not on the temperature variation rate but on the temperature difference. The measured pyroelectric coefficient depended on the sample temperature, and had a maximum value of about 6.97 × 10^−4^ C/m^2^ K. A distribution of the calculated pyroelectric coefficient with the related data of temperature and current is shown in Figure 13. Obviously, the pyroelectric coefficient revealed a performance comparable to that in the literature [14,15]. Moreover, increasing the electric output still needed to simultaneously support the absorption coefficient, the pyroelectric coefficient, the sensing area and the temperature variation rate. When the pyroelectric materials and dimensions were decided upon, the absorption coefficient, the pyroelectric coefficient and the sensing area were not altered. However, the temperature variation rate allows for greater freedom in designing and manufacturing patterns, trenches, cavities and structures in pyroelectric materials.

Table 3 summarizes the generated charge (Q), the generated charge per unit area (P_s_), the measured current (I_diff_) and the measured voltage (V_diff_) for pyroelectric cells with a fully covered electrode, as compared with a branch-like or vortex-like electrode in the PZT sheets. P_s_ can be defined as the magnitude of the electrical polarization vector [2,10]. The charge was inferred from the integration of the positive area added to the negative area enclosed under the current curves. Although the currents in both areas were inverse, a bridge rectifier circuit could be used to store the serviceable charges. The integral was estimated numerically using the trapezoid rule. Because pyroelectric energy harvesting requires temporal temperature variations, namely the cycles of heating and cooling, both the forward and the backward currents are useful for storing the charge by a bridge rectifier circuit. Therefore, Idiff is defined as the difference between the maximum forward and the minimum backward current. Then, V_diff_ is also defined as the difference between the maximum forward and the minimum backward voltage. Both I_diff_ and V_diff_ could be effortlessly used to estimate the efficiency of pyroelectric harvesters under thermal cycles of heating and cooling, to reveal the integral thermal property of the pyroelectric cells during the rising and falling temperature.

**Figure 14.**
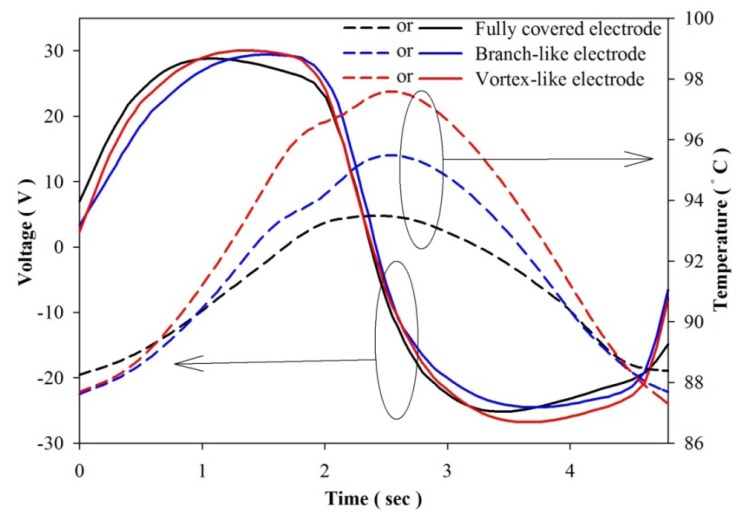
Measured values of voltage and temperature over time for various designs.

**Figure 15.**
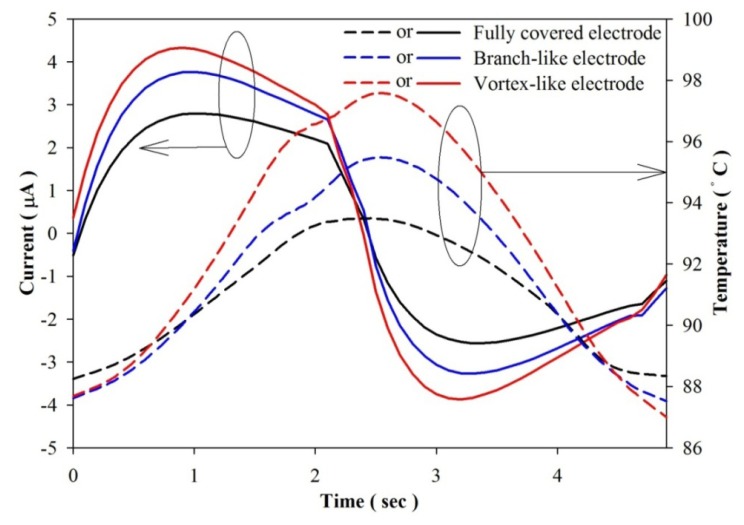
Measured values of current and temperature over time for various designs.

Figure 14 shows the measured values of voltage and temperature over time for the fully covered, the branch-like and the vortex-like electrodes. The voltage for the various designs exhibited no obvious differences. Figure 15 shows the measured values of current and temperature over time for the electrodes. The temperature in the PZT sheets had an undulation, as shown in Figures 14 and 15, due to the mobile platform working between the digital electronic hot air guns. The measured electrical output of the vortex-like electrode had an obvious increase on Q, P_S_ and I_diff_, compared to the fully covered electrode of about 47.1%, 47.1% and 53.1%, respectively. Based on Equation (1), the pyroelectric current was closely related to the temperature variation rates. The vortex-like electrode with a deep trench structure enhanced the temperature variation rates with lateral temperature gradients induced on the sidewalls of the trenches, which improved the electrical output of the pyroelectric cells and improving the efficiency of pyroelectric harvesting converters. Nevertheless, the measured electrical output of the branch-like electrode had a light increase on Q, P_S_ and I_diff_, compared to the fully covered electrode of about 29.3%, 29.3% and 31.3%, respectively. Although a constant heat mode and frequency was used by the digital hot air gun with the mobile platform and the step motor controlled by the pulse controller, the temperature in the fully covered electrode during the repetition of heating and cooling was lower than that in the branch-like or the vortex-like electrodes because of a high thermal capacity and an empty design for degrading the heat absorption in the fully covered electrode. The two patterned PZT pyroelectric sheets had the same projected trenched electrode area of about 130 mm^2^ and top electrode area of about 194 mm^2^. Hence, the two patterned PZT pyroelectric sheets had the same heat capacity. When the PZT pyroelectric sheets were measured for temperature fluctuations from 50 °C to 210 °C with a period of 4 s, the range in the fully covered, branch-like and vortex-like types was 5.2 °C, 7.9 °C and 10.7 °C, respectively. Apparently, the vortex-like electrode could rapidly raise and reduce the temperature in the PZT sheet because it possessed a low thermal capacity, and the vortex-like electrode and trench design enhanced the heat absorption. This helped the generation of electrical energy by pyroelectricity. The results from the experiments were consistent with those from the simulations. The vortex-like type compared to the fully covered type had a 53% increased range in the temperature variation rate, which was almost the same as that in the measured current. Moreover, the branch-like type also had a lower temperature variation rate and electrical output compared to the vortex-like type. The higher temperature variation rate in the vortex-like type, compared to the branch-like type, was mainly at the points diverging from the center of the PZT sheets.

**Figure 16.**
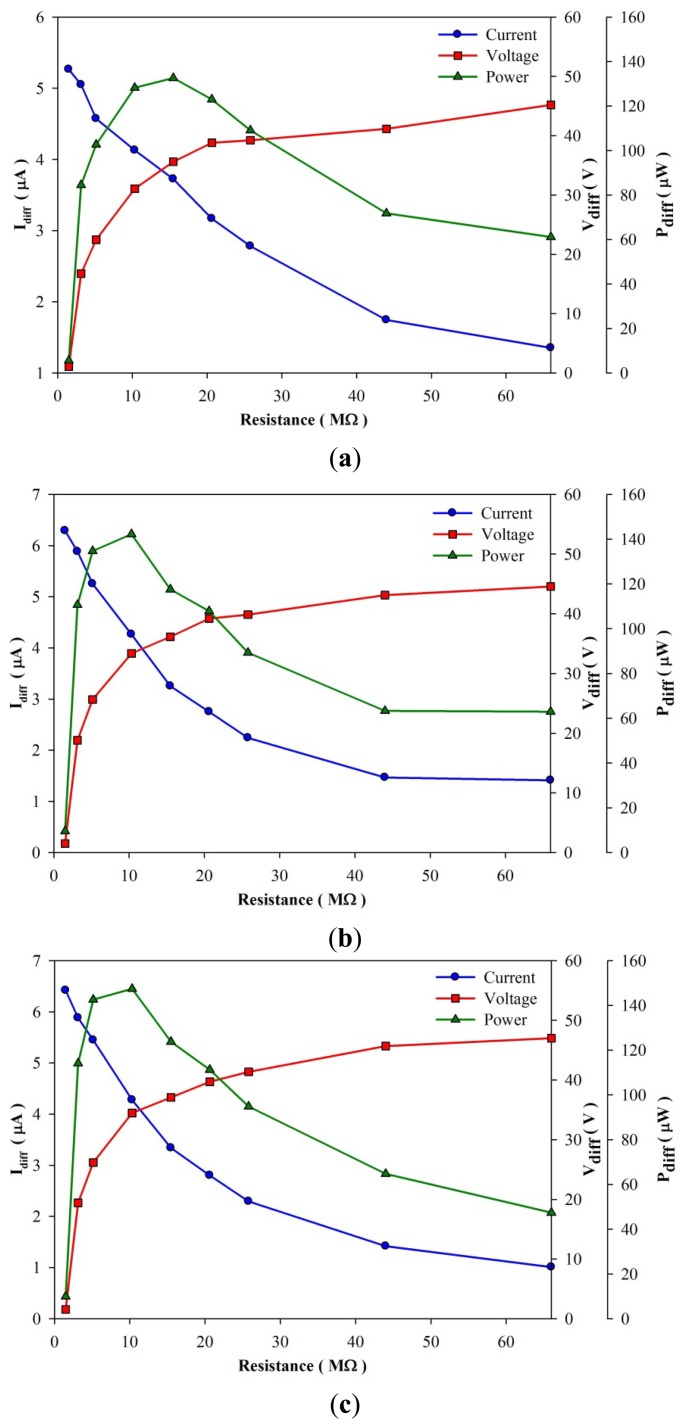
Harvested power as function of load resistance (R_L_) in various designs for the (**a**) fully covered; (**b**) branch-like and (**c**) vortex-like electrodes.

**Figure 17.**
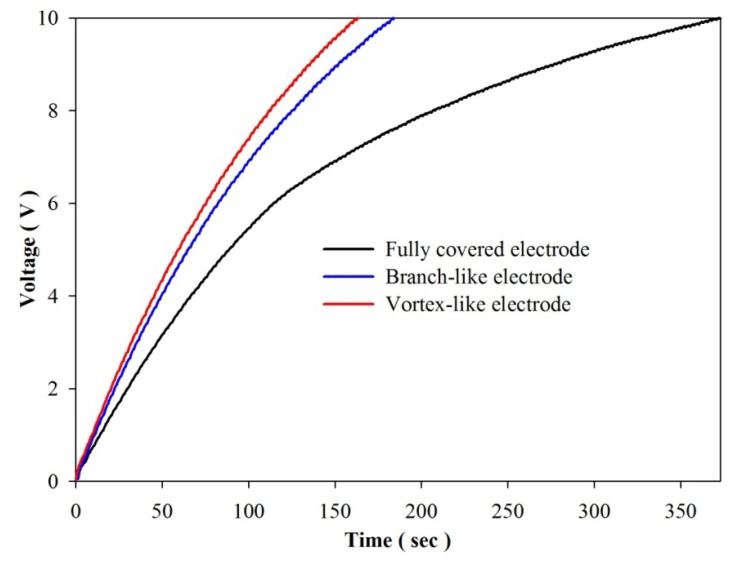
V_0_ across the capacitor (C_L_) as a storage element over time during consecutive heating and cooling cycles for various designs.

Figure 16 shows the harvested power as a function of load resistance (R_L_) in the various designs for the fully covered, branch-like and vortex-like types. P_diff_ is defined as the difference between the maximum forward and the minimum backward power. P_diff_ of the vortex-like type was about 11.3% higher than that of the fully covered type, while P_diff_ of the branch-like type was a mere 7.4% higher. The optimal load resistance in the various designs was about 10∼15 MΩ. Although the two patterned PZT pyroelectric sheets had the same trenched electrode area of about 130 mm^2^, various patterns affected the electrical yields under the constant heat mode and frequency used. The vortex-like type performed better than the branch-like type did, which could be attributed to the trenches in the branch-like type's scattering heat absorption. The heat source supplied for the pyroelectric cells to produce electrical yields was convective heat transfer by heated air. The branch-like type could guide the rapid flow of heated air out of the pyroelectric sheets. However, the vortex-like type caught the heated air within the pyroelectric sheets, which promoted heat absorption. For further proof of the predominance of the vortex-like type, the stored energy increased and the voltage V_L_ across C_L_, as depicted in Figure 17. When C_L_ was charged to a voltage of about 10 V, the vortex-like type merely expended heat for about 163 s; the branch-like and the fully covered type were 184 s and 375 s, respectively. The vortex-like and the branch-like type economized on time by about 2.3 and 2 times, respectively, over the fully covered type. Therefore, various patterns in pyroelectric sheets greatly affected the efficiency of pyroelectric harvesting converters. The sandblast etching apparatus successfully manufactured the complex patterns with the deep trench structures. A complex pattern with a deep trench structure designed for a specific heat mode or type could easily be accomplished with the sandblast etching method.

## 5. Conclusions

This paper reported a three-dimensional pattern of the vortex-like electrode in the PZT pyroelectric cells fabricated by a novel sandblast etching method for improving the electrical output, and enhancing the efficiency of PZT pyroelectric harvesting converters. Creating a complex pattern with a deep trench structure for fitting various distributions, densities, magnitudes and intensities of waste heat sources was necessary to ameliorate the thermal energy harvesting, but hard to fabricate using the traditional methods of dry/wet etching and a dicing saw. The results from both the experiment and simulation showed a positive enhancement in the proposed design of the vortex-like electrode for improving the electrical output of PZT pyroelectric harvesting converters. In the simulation results, the vortex-like type has a larger temperature variation rate than the fully covered type, about 53.9%. Moreover, the measured electrical output of the vortex-like electrode shows an obvious increase on the generated charge, the measured current and the harvested power, compared to the fully covered electrode, of about 47.1%, 53.1% and 11.3%, respectively.

## Figures and Tables

**Table 1.** Properties of the commercial PZT pyroelectric sheet.

**Sample** **ID**	**Thickness** **(μm)**	**Area** **(mm^2^)**	**Size** **(mm × mm)**	**Relative Dielectric Constant (ε_33_^T^/ε_O_)**	**Density (g/cm^3^)**	**Poling Field (V/μm)**	**Pyroelectric Coefficient (10^−4^ C/m^−2^·K^−1^)**
KA	200	324	18 × 18	2,100	7.9	3.5	6.97

**Table 2.** Material parameters used for finite element analysis.

**Material**	**Thermal Conductivity** **(W m^−1^ K^−1^)**	**Specific Heat** **(J g^−1^ K^−1^)**	**Density** **(g cm^−3^)**	**Dimension (mm)**
PZT sheet	2.1	0.36	7.9	18 × 18 × 0.2
Air	−0.00227583562 + 1.15480022E-4 × T − 7.90252856E-8 × T^2^ + 4.11702505E-11 × T^3^ − 7.43864331E-15 × T^4^	1.04763657 − 3.72589265E-4 × T + 9.45304214E-7 × T^2^ − 6.02409443E-10 × T^3^ + 1.2858961E-13 × T^4^	101.325 × 10^3^ × 0.02897/ 8.314/T	25 × 25 × 25

**Table 3.** Measured electrical outputs for the pyroelectric sheets.

**PZT Sample**	**Fully Covered** **Electrode**	**Branch-like** **Electrode**	**Vortex-Like** **Electrode**
Top electrode area (mm^2^)	324	194	194
Projected trench electrode area (mm^2^)	non	130	130
Q (μC)	9.9	12.9	14.7
P_s_ (10^−2^ C/m^2^)	3.0	3.9	4.6
I_diff_ (μA)	5.3	7.1	8.2
V_diff_ (V)	53.9	53.9	56.9
W_max_ (μW)	132.5	142.2	147.5
